# Shared and distinct retinal input to the mouse superior colliculus and dorsal lateral geniculate nucleus

**DOI:** 10.1152/jn.00227.2016

**Published:** 2016-05-11

**Authors:** Erika M. Ellis, Gregory Gauvain, Benjamin Sivyer, Gabe J. Murphy

**Affiliations:** ^1^Janelia Research Campus, Howard Hughes Medical Institute, Ashburn, Virginia; and; ^2^Queensland Brain Institute, The University of Queensland, St Lucia, Queensland, Australia

**Keywords:** retina, retinorecipient, superior colliculus, thalamus

## Abstract

The mammalian retina conveys the vast majority of information about visual stimuli to two brain regions: the dorsal lateral geniculate nucleus (dLGN) and the superior colliculus (SC). The degree to which retinal ganglion cells (RGCs) send similar or distinct information to the two areas remains unclear despite the important constraints that different patterns of RGC input place on downstream visual processing. To resolve this ambiguity, we injected a glycoprotein-deficient rabies virus coding for the expression of a fluorescent protein into the dLGN or SC; rabies virus labeled a smaller fraction of RGCs than lipophilic dyes such as DiI but, crucially, did not label RGC axons of passage. Approximately 80% of the RGCs infected by rabies virus injected into the dLGN were colabeled with DiI injected into the SC, suggesting that many dLGN-projecting RGCs also project to the SC. However, functional characterization of RGCs revealed that the SC receives input from several classes of RGCs that largely avoid the dLGN, in particular RGCs in which *1*) sustained changes in light intensity elicit transient changes in firing rate and/or *2*) a small range of stimulus sizes or temporal fluctuations in light intensity elicit robust activity. Taken together, our results illustrate several unexpected asymmetries in the information that the mouse retina conveys to two major downstream targets and suggest that differences in the output of dLGN and SC neurons reflect, at least in part, differences in the functional properties of RGCs that innervate the SC but not the dLGN.

## Abstract (precis)

*Our results suggest that the mouse superior colliculus (SC) has access to input from most of the retinal ganglion cells (RGCs) that innervate the dorsal lateral geniculate nucleus (dLGN). By comparison, a number of RGC types appear to innervate the SC but not the dLGN; these RGCs generally exhibit more transient responses and respond best to small stimuli*.

## NEW & NOTEWORTHY

*Our results suggest that the mouse superior colliculus (SC) has access to input from most of the retinal ganglion cells (RGCs) that innervate the dorsal lateral geniculate nucleus (dLGN). By comparison, a number of RGC types appear to innervate the SC but not the dLGN; these RGCs generally exhibit more transient responses and respond best to small stimuli*.

different regions of the brain represent information about overlapping but distinguishable features of visual stimuli. Neurons in the dorsal stream of the primate visual system, for example, frequently encode information about object motion, whereas neurons in the ventral stream often encode information about chromatic and/or spatial characteristics (Livingstone and Hubel 1988; Maunsell and Newsome 1987; see, however, Merigan and Maunsell 1993). Neurons in distinct areas of the rodent visual system exhibit similar functional differences (Andermann et al. 2011; Garrett et al. 2014; Marshel et al. 2011).

Parallel processing is not restricted to visual cortical areas. Indeed, although some neurons in the mouse dorsal lateral geniculate nucleus (dLGN) and superior colliculus (SC) encode information about the same features of visual stimuli, e.g., orientation or motion direction (Ahmadlou and Heimel 2015; Feinberg and Meister 2015; Marshel et al. 2012; Zhao et al. 2013), most dLGN and SC neurons respond best to distinct characteristics of visual stimuli. The overwhelming majority of dLGN neurons in rodents, as in primates, exhibit circularly symmetric receptive fields, a strong preference for either increases or decreases in light intensity, and linear spatial summation (Denman and Contreras 2015; Grubb and Thompson 2003, 2005; Piscopo et al. 2013). Neurons in the superficial (retinorecipient) layers of the rodent SC, on the other hand, frequently exhibit irregularly shaped receptive fields, sensitivity to both increases and decreases in light intensity, and/or responses to spatiotemporal patterns of light stimuli that are not predictable from the linear summation of responses to small, stationary stimuli (Drager and Hubel 1975; Gale and Murphy 2014; Humphrey 1968; Mooney et al. 1988).

To what extent, if at all, might differences in the output of dLGN and SC neurons reflect differences in the retinal input they receive? One hypothesis is that dLGN and SC neurons exhibit distinct outputs despite receiving input from similar sets of retinal ganglion cells (RGCs); indeed, distinct local processing of shared retinal input might be necessary because most (of the small number of) reconstructed rodent and cat RGC axonal arbors innervate both the dLGN and SC (Bowling and Michael 1980; Dhande et al. 2011). An alternative hypothesis, however, is that differences in the output of dLGN and SC neurons reflect differences in the retinal input the neurons receive (de Monasterio 1978; Rodieck and Watanabe 1993; Schiller and Malpelli 1977). This is plausible because substantially more RGCs innervate one retinorecipient target than the other (Dreher et al. 1985; Linden and Perry 1983; Martin 1986; Perry et al. 1984).

Identifying the shared and distinct information that two brain regions receive is important; it pinpoints where neural circuits extract information about specific features of sensory stimuli. Identifying shared and distinct sources of input is also difficult; in the early visual system it requires *1*) robust and specific labeling and *2*) functional characterization of populations of SC-, dLGN-, and SC + dLGN-projecting RGCs. We satisfied these requirements by determining the fraction of mouse RGCs labeled via injection of rabies virus and/or retrogradely transported fluorescent compounds into the SC and/or dLGN; subsequent electrophysiological recordings enabled quantitative characterization and comparison of the light response properties of labeled RGCs. This approach revealed that many mouse RGCs innervating the dLGN also innervate the SC; the same approach also revealed, however, that the functional characteristics of RGC input to the SC and dLGN differ in several important ways.

## MATERIALS AND METHODS

### 

#### Mice, reagents, and surgical procedures.

Experiments were performed on 5- to 12-wk-old c57bl6J mice of either sex. All procedures were approved by the Institutional Animal Care and Use Committee at the Janelia Research Campus. A subset of experiments used transgenic mice: vGlut2-Cre (Vong et al. 2011), Ai14 (Madisen et al. 2010), and Hb9::eGFP (Arber et al. 1999; Trenholm et al. 2011).

We used stereotaxic injection of glycoprotein (G)-deleted rabies virus and/or DiO/DiI/DiD (Life Technologies, Grand Island, NY) to retrogradely label RGCs from the SC and/or dLGN. Rabies virus was generated by the molecular biology facility at the Janelia Research Campus; the virus drove expression of green fluorescent protein (GFP) or mCherry. Exactly the same rabies virus was used to infect the axon terminals of RGCs in the dLGN and SC, ruling out the possibility that observed differences in the functional characteristics of SC- and dLGN-projecting RGCs were a consequence of viral tropism.

**Fig. 1.**
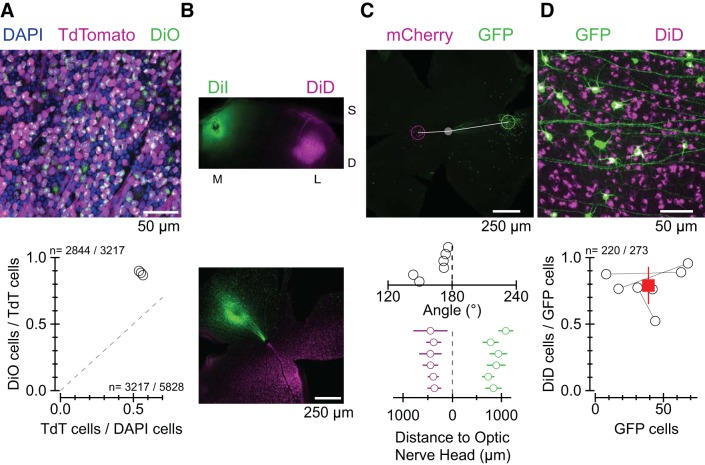
The vast majority of RGCs, and the vast majority of dLGN-projecting RGCs, innervate the SC. *A*: 88% of mouse RGCs are retrogradely labeled from the SC. *Top*, confocal image of the ganglion cell layer of vGlut-Cre × Ai14 mouse retina ∼7 days after ∼100 nl of DiO were injected into the SC. DAPI (blue) labeled all cells, TdTomato (purple) is expressed in RGCs, and DiO (green) labels RGCs retrogradely labeled from the SC. *Bottom*, each circle represents, for a given retina, the fraction of RGCs labeled with DiO (*y*-axis) vs. the fraction of ganglion cell layer cells expressing TdTomato (*x*-axis). *B*, *top*: fluorescence image of an SC in which an unusually small (<10 μl) volume of DiI was injected medially (M) and a small volume of DiD was injected laterally (L). S and D denote the superficial and deep SC, respectively. *Bottom*, fluorescence image of the retina contralateral to the SC shown above. *C*, *top*: fluorescence image of a retina from an animal in which ΔG-Rabies_mCherry and ΔG-Rabies_GFP were injected into the rostral and caudal SC, respectively. Gray circle denotes the location of the optic nerve head; purple and green circles denote 2D Gaussian fits to the channels capturing mCherry and GFP fluorescence emission, respectively. The angle separating the center of the 2D Gaussian fits to the mCherry and GFP fluorescence emission in each of 6 eyes is shown at *bottom*. *Bottom*, the absolute distance from the optic nerve head to the center of the two 2D Gaussian fits in each of the 6 eyes; error bars indicate 2 times the standard deviation of the fits. *D*: many dLGN-projecting RGCs also innervate the SC. *Top*, confocal image of an area of retina from an animal in which ΔG_Rabies-GFP was injected into the dLGN (green) and DiD (∼100 nl) was injected into the SC (purple). *Bottom*, each circle represents, for a given retina, the fraction of dLGN-projecting RGCs that were also DiD positive (*y*-axis); lines link data obtained from the 2 eyes of a given mouse. The red square and error bars represent the mean ± SE across 7 retinas. Note that there were many GFP-positive cells in areas of the retina that exhibited sparse/weak DiD labeling; these RGCs/areas were excluded from analysis.

Stereotaxic coordinates for SC injections were 0–0.5 mm rostral from lambda, 0.3–1.0 mm lateral from the midline, and 0.9–1.2 mm deep from the pial surface. Coordinates targeting the dLGN were 1.2–1.6 mm rostral to lambda, 2.2–2.3 mm lateral to the midline, and 2.5–2.7 mm below the pial surface. In nearly all cases we injected 50–100 nl of virus or DiO/DiI/DiD along each injection tract. We regularly ejected reagents at three different depths along a tract (each injection site was separated by at least 50 μm) to label RGC axons distributed throughout the dorsal ventral axis of a given retinorecipient area. We did this for two reasons. First, from a practical point of view, there is little consequence of injecting too deep. Second, and perhaps more importantly, we thought it important to distribute reagents throughout the “shell” and the “core” of the dLGN and through the entire depth of the retinorecipient layers of the SC. We injected a small volume (<10 nl) of a given reagent at only one depth in a small subset of experiments (shown in Fig. 1*B*).

Experiments were performed >6 days after injection of DiO/DiI/DiR and 4–6 days after injection of rabies virus. In cases in which both reagents were injected into the same animal, DiO/DiI/DiR was injected into the SC first (because transport of DiO/DiI/DiR from the SC takes longer than virus-mediated expression of fluorescent proteins). Three to 5 days later, rabies virus was injected into the dLGN; retinal tissue was extracted 4–6 days after rabies had been injected into the dLGN.

After dark-adapting overnight, animals were killed via cervical dislocation; this and all subsequent procedures were performed under infrared (>900 nm) illumination. The eyes were dissected and stored in a light-tight container in bicarbonate-buffered Ames solution (Sigma-Aldrich, St. Louis, MO) equilibrated with carbogen (95% O_2_-5% CO_2_). The cornea, lens, and vitreous were removed mechanically from each eye. Subsequently, a section of the eye cup was cut with a scalpel blade and the retina isolated from the pigment epithelium. The retina was placed ganglion cell side up on a piece of filter paper (Anodisc13; Whatman); the filter paper was then secured to a glass-bottom recording chamber via grease. The retina was held to the filter paper via nylon wires stretched across a platinum scaffold. Warm (30–34°) equilibrated Ames solution perfused the recording chamber at a rate of ∼6 ml/min.

#### Multiphoton excitation and confocal scanning microscopy.

Retrogradely labeled RGCs in living tissue were identified via multiphoton excitation fluorescence laser scanning microscopy (Ultima; Prairie Technologies). The Ti:sapphire laser (Ultra II; Coherent) was tuned to ∼900 ± 20 nm. The laser intensity was modulated by a Pockel's cell (Conoptics); laser intensity at the preparation was <10 mW. The laser was used only to identify fluorescent cells from which to record electrophysiological signatures; the laser was shuttered while we recorded responses of RGCs to visual stimuli.

Images of fixed tissue were acquired using a confocal microscope (LSM-700; Carl Zeiss, Oberkochen, Germany) outfitted with a ×20, ×40, or ×63 objective; most images were composed of 1,024 × 1024 pixels. Images were stitched together, when necessary and appropriate, via Zen software (Carl Zeiss) or custom routines written in MATLAB (The MathWorks). Labeled cells were counted manually using the FIJI cell counter plugin (NIH; http://fiji.sc/Fiji).

#### Light stimuli and collection and analysis of electrophysiological data.

Stimuli were specified via custom software (written in openGL by Anthony Leonardo and Calin Culineau) and generated via a customized DMD projector (DepthQ; InFocus) with a refresh rate of 120 Hz. The output of the DMD 1,280 × 780 array was delivered to the recording chamber via a dichroic mirror placed beneath the condenser lens of an upright microscope (Zeiss); each pixel was ∼1 μm^2^. The light source, a broad-spectrum white LED (SugarCUBE; Nathaniel Group, Vermont), was attenuated by a series of neutral density filters. The maximum intensity of the light stimulus at the preparation was ∼3 × 10^6^ photons·μm^−2^·s^−1^. The background light level delivered during most experiments was 1–10% of the maximum intensity; i.e., well into the “photopic” range.

Cell-attached (or “loose seal”) patch-clamp recordings were performed with pipettes fabricated from thin-wall borosilicate glass (Sutter). We filled the pipettes (4–8 MΩ) with Ames solution. Electrophysiological signals were amplified and then low-pass filtered at 3–5 kHz (Multiclamp 700B; Molecular Devices); data were digitized at 10 kHz (ITC-18; HEKA) and saved to disk via custom, open-source software (Symphony; https://github.com/Symphony-DAS/symphony/). GFP- or mCherry-labeled cells exhibited a range of soma sizes; we did not find it especially difficult to record from smaller cells (in part because cell-attached recordings are less demanding than whole cell recordings). Moreover, we did not observe any obvious differences in the response properties of nearby rabies-infected and rabies-free *1*) “alpha-like” or *2*) On-Off direction-selective RGCs.

Electrophysiology data reported in this article were obtained from a total of 28 mice; 6–7 cells were characterized, on average, from each mouse (range 1–28 cells). Analyses of light-evoked responses were performed via procedures written in MATLAB. Light-evoked responses were aligned to stimulus onset via measurement (with a photodiode) of a signal that changed with each frame; spike times, once aligned to the time of stimulus onset, were used to generate a spike density function (SDF; Szucs 1998).

We began each experiment by determining how the cell's response varied as a function of stimulus contrast and duration; we then assayed the response of the cell to five stimulus sizes (50–800 μm) using the stimulus contrast that elicited slightly submaximal responses. Thereafter, using the stimulus size that produced the maximal response, we assayed the response of a cell to stimuli moving in each of 8 directions at a given speed of 800 μm/s. Finally, we moved the same-size stimulus in a cell's preferred direction at seven to eight different speeds (that ranged from 50 to 3,200 μm/s).

Direction selectivity was computed as the vector sum of responses (to stimuli moving in 8 different directions) normalized by the scalar sum of responses; the amplitude of the normalized vector sum length (NVSL) varies between 0 and 1, and the angle of the NVSL defined the preferred direction of each cell. All dLGN-projecting On-Off direction-selective RGCs and 19/20 SC-projecting On-Off direction-selective RGCs had a NVSL value >0.2. We did not always determine the orientation of the eye or the piece of retina cut from it; it was therefore impossible to determine the eye- or head-centric coordinates to which direction- or orientation-selective RGCs responded best.

Response duration is the integral of the SDF over a 500-ms window starting at the response latency (>0.1 × peak response for at least 50 ms) divided by the peak response and window duration. A cell's “best size” reflects the stimulus size that resulted in the largest peak response; best size fit is the same for a gamma function fit (Nover et al. 2005) of the size tuning curve. Size tuning width and speed tuning width are the normalized area (integral/peak) of the size tuning and speed tuning curve, respectively. Size tuning and speed tuning fit are the same for a gamma function fit of the appropriate relationship.

## RESULTS

### 

#### Eighty-five to ninety percent of mouse RGCs project to the SC.

To estimate the fraction of RGCs that project to a given retinorecipient area requires one to first identify RGCs. This is nontrivial because the ganglion cell layer of the mammalian retina contains both RGCs and a substantial number of amacrine cells, i.e., GABA, and/or glycinergic interneurons that do not project to the brain.

Many RGCs, and few if any amacrine cells, express vesicular glutamate transporter 2 (vGlut2; Johnson et al. 2003; Land et al. 2004; Sherry et al. 2003). Therefore, to label RGCs selectively, we crossed transgenic mouse lines in which Cre recombinase is expressed in vGlut2-expressing cells (Vong et al. 2011), and the red fluorescent protein TdTomato is expressed in cells that express Cre recombinase (Ai14; Madisen et al. 2010). SC-projecting RGCs were identified by injecting retrogradely transported lipophilic fluorescent compounds such as DiO into the SC; as in our previous work (Gauvain and Murphy 2015), DiO was targeted to the caudal half of the SC to avoid labeling retinorecipient nuclei just rostral to the SC.

Retinal tissue collected 7+ days after DiO was injected into the SC of vGlut2-Cre × Ai14 animals supports several conclusions. First, because DiO only labeled TdTomato-positive cells, many, if not all, RGCs express TdTomato in vGlut2-Cre × Ai14 animals; we would expect to see DiO in cells lacking TdTomato otherwise. Second, because DiO never labeled a cell lacking TdTomato, the absence of TdTomato almost certainly indicates that a cell is an amacrine cell. Third, RGCs represent a slight majority of cells in the mouse ganglion cell layer; an average of 55% of all ganglion cell layer cells expressed TdTomato (Fig. 1*A*, *bottom*, *x*-axis; range 53.8–56.6% across 3 retinas from 3 mice). Fourth, the vast majority of mouse RGCs innervate the SC; in areas of the retina densely labeled from the SC, ∼88% of TdTomato-positive cells were also DiO positive (in each of the same 3 retinas: Fig. 1*A*, *bottom*, *y*-axis; range 86.7–88.9%).

#### Approximately 80% of dLGN-projecting RGCs also innervate the SC.

The axons of SC-projecting RGCs pass through the dLGN (see, for example, Morin and Studholme 2014). To identify RGCs that innervate the dLGN but not the SC, as well as the fraction of dLGN-projecting RGCs that send an axon collateral to the SC, requires one to use reagents that label cells that synapse in the dLGN without labeling cells that simply send an axon through/near the same area; commonly used reagents such as DiO/DiI, horseradish peroxidase, or fluorophore-conjugated latex microspheres or cholera toxin are not sufficient/suitable.

Several reports suggest that rabies virus is taken up preferentially if not exclusively at presynaptic terminals (reviewed in Callaway and Luo 2015; Ugolini 2010). To test this hypothesis, we injected a G-deleted rabies virus (Wickersham et al. 2007) coding for the expression of mCherry into the rostral SC and the same virus coding for expression of GFP into the caudal SC. If the virus does not infect axons of passage, then *1*) GFP- and mCherry-expressing RGCs should occupy separate areas of the retina, because RGCs innervating the rostral and caudal SC are located in the temporal and nasal retina, respectively (Siminoff et al. 1966; Simon and O'Leary 1992); and *2*) RGCs innervating the caudal SC should never express mCherry despite sending an axon through the rostral SC.

We found that GFP- and mCherry-expressing RGCs nearly always occupied opposite sides of the retina (Fig. 1*C*, *top*). Indeed, ∼164° separated the vectors linking the optic nerve head to the center of two-dimensional (2D) Gaussian fits to GFP- and mCherry-expressing RGC populations (*n* = 6 eyes from 3 animals; Fig. 1*C*). The absolute distance between the centers of the two populations of RGCs (1,757 ± 101 μm) was an order of magnitude larger than the standard deviation of the 2D Gaussian fit to either population (Fig. 1*C*, *bottom*), and no GFP-expressing cells were within two standard deviations of the center of the area occupied by mCherry-expressing cells. Moreover, mCherry-expressing RGCs were always tightly clustered together on the temporal side of the optic nerve head; i.e., they did not occupy (nasal) areas of the retina densely labeled from the caudal SC. Together, these results indicate that G-deleted rabies virus is unlikely to infect RGC axons of passage.

The results of experiments shown in Fig. 1*C* indicate that rabies virus can be used to infect RGCs that synapse in the dLGN without labeling axons that simply pass through the region on their way to the SC. Therefore, to estimate the fraction of dLGN-projecting RGCs that innervate the SC, we first injected DiI or DiD bilaterally into the caudal SC of a given animal. Three to 5 days later we injected rabies virus coding for the expression of GFP bilaterally into the dLGN of the same animal; reagents were injected in both regions via multiple tracts and at multiple depths to *1*) label RGCs axons at different planes along the dorsal-ventral axis, *2*) maximize the number of labeled RGCs, and *3*) maximize the retinal area in which RGCs exhibited GFP and DiI/DiD labeling.

After waiting 4–6 days for GFP expression, we quantified (in areas of the retina densely labeled from the SC) the fraction of GFP-expressing cells that also exhibited DiI/DiD; focusing on the areas most densely labeled with DiI or DiD increased the confidence with which we could interpret the absence of retrograde label in an RGC as evidence that the RGC did not project to the SC (see Fig. 1*A*). We found that DiI/DiD labeled, on average, >80% of GFP-expressing RGCs (range: 52–96% across 7 retinas from 4 animals; Fig. 1*D*). Thus most, but not all, of the information that the retina conveys to the dLGN is also sent to the SC.

#### Functional characterization and comparison of On-Off direction-selective RGCs retrogradely labeled from the dLGN and SC.

The data presented in Fig. 1 do not, on their own, resolve the degree to which the retina sends similar or distinct information to the dLGN and SC. The ∼20% of dLGN-projecting RGCs that do not extend an axon collateral to the SC might, for example, be functionally distinct from the ∼80% that do. Moreover, anatomical information indicates that some mouse RGCs synapse within the SC but not the dLGN (Dhande et al. 2011); the number and functional properties of such “SC only” RGCs are unknown.

We reasoned that the response properties of RGCs labeled from the dLGN and SC should be similar if most RGCs innervate both the dLGN and SC; by the same logic, the response properties of RGCs labeled from the dLGN and SC should be different if *1*) a significant fraction of RGCs innervate one area but not the other and *2*) RGCs targeting only one area respond to distinct/specific subsets of stimulus characteristics. To test these hypotheses, we identified RGCs infected from the SC or dLGN via multiphoton fluorescence microscopy and recorded the responses of these cells to a number of visual stimuli. In each of 192 labeled RGCs, we assayed how action potential generation varied as a function of stimulus contrast, size, duration, direction and speed of motion, and temporal modulation of luminance; this information enabled us to distinguish several functional classes of mammalian RGC (reviewed in Sanes and Masland 2015).

**Fig. 2.**
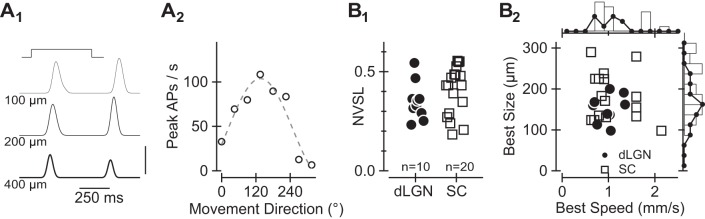
Functional characteristics of On-Off direction-selective (DS) RGCs retrogradely labeled from the dLGN or SC. *A*_*1*_: average response of a representative On-Off DS RGC to the presentation of a bright spot with a diameter of 100, 200, or 400 μm. Vertical scale bar at *bottom right* represents 100 action potentials (APs)/s. *A*_*2*_: average rate of AP generation of the same cell to movement of a 200-μm spot (through the center of the cell's receptive field) in 8 different directions at 800 μm/s, i.e., ∼30°/s. *B*_*1*_: the degree of direction selectivity (quantified via the normalized vector sum length, NVSL) in each cell. *B*_*2*_: the interpolated best stimulus size (*y-*axis) and interpolated best stimulus velocity (*x-*axis) for each of the On-Off DS RGCs labeled from the dLGN (filled circles) and SC (open squares). Histograms formed by separately collapsing values along the *x*- and *y*-axes and then binning are shown at *right* and *top*, respectively; open bars summarize the SC data whereas lines connecting filled circles summarize the LGN data.

Figure 2*A* shows the response of a SC-projecting RGC to *1*) a bright circle (of three different diameters) on a dark background and *2*) different directions of movement of the stimulus size that produced a maximal response. The cell responds to the onset and offset of the stationary light stimulus (Fig. 2*A*_*1*_) and strongly prefers stimuli moving in a subset of directions (Fig. 2*A*_*2*_).

This cell exhibits most, if not all, of the characteristics of one of the most readily identified/studied classes of mammalian RGC; i.e., On-Off direction-selective (DS) RGCs (reviewed in Vaney et al. 2012). As predicted from previous results (Huberman et al. 2009; Kay et al. 2011; Kim et al. 2010), we found that On-Off DS RGCs were retrogradely labeled from the dLGN and SC. However, On-Off DS RGCs comprised a substantially larger fraction of the RGCs retrogradely labeled from the SC (20/103; Fig. 2*B*_*1*_, *right*) than from the dLGN (10/89; Fig. 2*B*_*1*_, *left*). We found limited evidence for projection-specific differences in the functional properties of On-Off DS RGCs (Kay et al., 2011; Rivlon-Etzion et al. 2011); dLGN- and SC-projecting On-Off DS RGCs exhibited similar degrees of direction selectivity (*P* > 0.3 via Wilcoxon-Mann-Whitney rank test; Fig. 2*B*_*1*_) and responded best to overlapping sizes and velocities of stimuli (*P* > 0.4 in both cases; Fig. 2*B*_*2*_). However, we noticed in this dataset and a previous one from our group (Gauvain and Murphy 2015) that a small but noticeable subset of SC-projecting On-Off DS RGCs respond best to unusually high stimulus speeds. Together, our results are most consistent with the idea that many individual On-Off DS RGCs innervate both the dLGN and SC, whereas some innervate only the SC.

#### Differences in retinal input to the dLGN and SC from Off and On RGCs.

On-Off DS RGCs represent only a subset of the total retinal input to the brain. We therefore performed a similar analysis for cells that responded to the offset of a light stimulus; i.e., Off RGCs. To distinguish different types of Off RGCs and compare the characteristics and relative abundance of these subtypes, we quantified the dynamics of action potential generation *1*) following a 500-, 1,000-, or 2,000-ms step increase in light intensity and *2*) in response to sinusoidal modulation of light intensity over time. This approach has helped distinguish functionally distinct groups of RGCs in a number of species, including mice (Baden et al. 2016; Farrow and Masland 2011; Margolis and Detwiler 2007; Murphy and Rieke 2006; Pang et al. 2003).

**Fig. 3.**
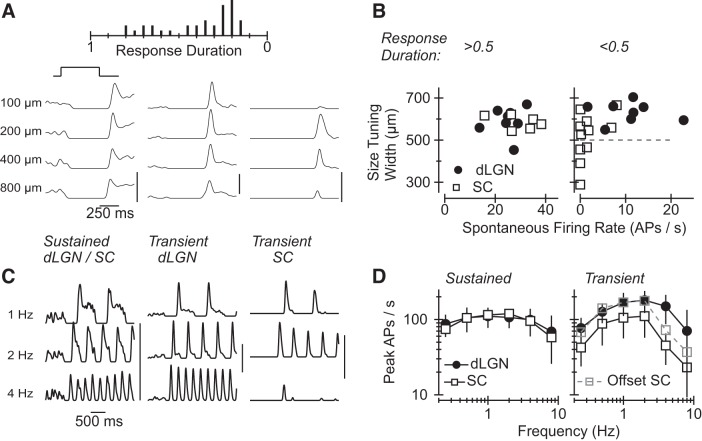
Functional characteristics of Off RGCs retrogradely labeled from the dLGN or SC. *A*: similarities and differences in the response properties of Off RGCs labeled from the dLGN and SC. The histogram at *top* shows the number of RGCs as a function of the RGCs' response duration; the *y*-axis is the cell count and the *x*-axis represents response duration values (binned in increments of 0.05). Responses of 3 representative RGCs to the presentation of a 100-, 200-, 400-, and 800-μm bright spot on a dark background are shown at bottom. Vertical scale bar in this and all subsequent panels represents 200 APs/s. *B*: the size tuning width vs. spontaneous firing rate for each Off RGC. Cells that exhibited sustained and transient changes in firing rate following a decrease in light intensity are shown at *left* and *right*, respectively. Data from RGCs labeled from the dLGN and SC are represented by filled circles and open squares, respectively. *C*: response of the same representative Off RGCs to 1-, 2-, and 4-Hz modulation of a large, spatially uniform spot of light. *D*: peak firing rate (±SD) as a function of stimulus frequency for sustained Off RGCs (*left*) and transient Off RGCs (*right*) labeled from the dLGN (solid line, filled circles) or SC (solid line, open squares). The dashed gray trace at *right* was formed by vertically shifting the SC data by 35 APs/s.

Off RGCs comprised a similar fraction of dLGN (19/89)- and SC (27/103)-projecting RGCs (*P* > 0.3 via χ^2^ test) and exhibited a broad diversity of response properties. One characteristic that varied between neurons was the degree to which neurons' responses were sustained or transient following an abrupt (“step”) decrease in light intensity (Fig. 3*A*). We found that Off RGCs exhibiting sustained changes in firing rate were encountered more frequently in the dLGN-projecting population of RGCs (11/19 vs. 8/27; *P* < 0.04 χ^2^ test). Like On-Off DS RGCs (Fig. 2), sustained Off RGCs exhibited similar functional characteristics whether they were labeled from the dLGN or SC; i.e., the spontaneous rate, response duration, and size tuning width were not significantly different between sustained Off RGCs labeled from the dLGN and SC (*P* > 0.25 in each case; Fig. 3*B*, *left*).

Off RGCs exhibiting sustained responses maintain elevated rates of action potential generation well after an abrupt decrease in light intensity (Fig. 3*A*, *left*) and during the phases of a sinusoidally modulated stimulus in which light intensity is low (Fig. 3*C*, *left*). Action potential generation returns to zero or near-zero values much more rapidly, by comparison, in Off RGCs exhibiting transient responses to the same stimuli (Fig. 3, *A* and *C*, *middle* and *right* columns). The fraction of Off RGCs exhibiting transient responses to light stimuli was similar in the SC (15/27)- and dLGN (8/19)-projecting populations of RGCs (*P* > 0.3, χ^2^ test). However, the ratio of transient to sustained Off RGCs was twice as large in the SC-projecting population of RGCs. To our surprise, dLGN- and SC-projecting Off transient RGCs exhibited significant functional differences. In particular, Off transient RGCs in the population retrogradely labeled from the dLGN generated more action potentials in darkness (Fig. 3*B*, *right*; 10.7 ± 6.3 vs. 1.8 ± 2.9; means ± SD; *P* < 0.001) and responded robustly to a larger range of stimulus sizes (*P* < 0.011). Indeed, 4/5 of the Off RGCs with size tuning widths <500 μm were retrogradely labeled from the SC (Fig. 3*D*); the stimulus size to which these cells responded best was generally, although not always, ≤200 μm. Moreover, the frequency at which responses to sinusoidal modulation of light intensity became half maximal, the corner “cutoff” frequency, was substantially lower in SC- than dLGN-projecting transient Off RGCs (Fig. 3, *C* and *D*). Together, these data suggest that the SC receives input from at least one class of transient Off RGC that does not innervate the dLGN; i.e., one that responds best to smaller stimuli and a smaller range of temporal fluctuations in light intensity. The same results suggests that Off Transient RGCs with moderate activity in darkness and broad size tuning are substantially more common in the dLGN- vs. SC-projecting pool of RGCs.

**Fig. 4.**
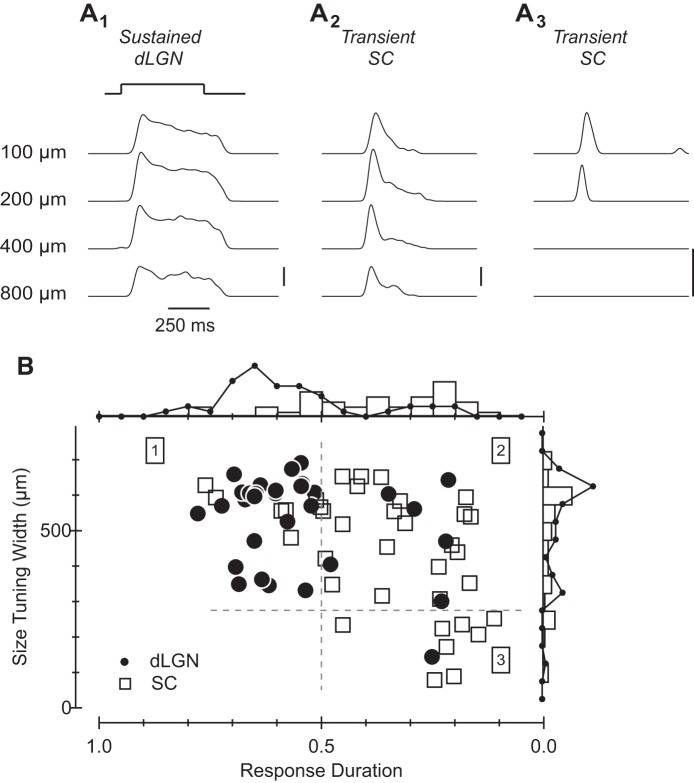
Functional characteristics of On RGCs retrogradely labeled from the dLGN or SC. *A*_*1*_: average response of a representative sustained On RGC to the presentation of a bright spot of increasing size; this cell, and many others like it, was retrogradely labeled from the dLGN. Vertical scale bar in this and all subsequent panels represents 100 APs/s. *A*_*2*_ and *A*_*3*_: same as in *A*_*1*_ for transient On RGCs (*A*_*2*_) and the group of RGCs that exhibits transient responses and selectivity for small stimuli retrogradely labeled from the SC (*A*_*3*_). *B*: scatter plot showing the response duration (*x*-axis) and size tuning width (*y*-axis) for each On RGC labeled from the dLGN (filled circles) and SC (open squares). Histograms formed by separately collapsing values along the *x*-and *y*-axes and then binning are shown at *right* and *top*, respectively; open bars summarize the SC data whereas lines connecting filled circles summarize the LGN data.

Several results indicate that On RGCs exhibit similar asymmetries. First, On RGCs comprised a substantially higher fraction of the RGCs labeled from the dLGN than SC (45/89 vs. 36/103; *P* < 0.03 via χ^2^ test). Second, as with Off RGCs, On RGCs exhibiting sustained responses were more common in the pool of dLGN-projecting RGCs, whereas those exhibiting transient responses were more common in the SC-projecting RGC population (*P* < 0.01; Fig. 4, *A* and *B*). Third, the population of RGCs labeled from the SC exhibited significantly narrower size tuning (*P* < 0.001). Indeed, 8 of the 9 cells that responded (transiently) only to small stimuli were in the SC-projecting population of RGCs (Fig. 4*A*_*3*_; *quadrant 3* of Fig. 4*B*). Collectively, our results suggest that the SC receives input from many of the types of RGC that innervate the dLGN in addition to several types that largely avoid the dLGN, e.g., Off and On RGCs that exhibit transient responses and/or respond to a limited range of (generally small) stimulus sizes.

## DISCUSSION

To what extent might differences in retinal input underlie differences in the output of dLGN and SC neurons? We find that SC neurons have access to much of the information the retina sends to the dLGN but not necessarily the converse; the same results suggest that RGCs that innervate the SC but avoid the dLGN frequently exhibit transient responses and/or sensitivity to small stimuli.

### 

#### Structure/anatomy.

A slight majority of cells in the ganglion cell layer of vGlut2-Cre × Ai14 mice expressed TdTomato. DiO injected into the SC of these animals never labeled a ganglion cell layer cell that lacked TdTomato; this *1*) argues against the possibility that a substantial fraction of RGCs in the retina of vGlut2-Cre × Ai14 animals lack TdTomato and *2*) indicates that many, if not all, amacrine cells lack TdTomato. On the other hand, the density of TdTomato labeling prevented us from determining whether each putative RGC had an axon; we cannot, therefore, rule out the possibility that TdTomato might be expressed spuriously in a small number of amacrine cells. Indeed, our estimate of the fraction of RGCs in the mouse ganglion cell layer, which is based on the fraction of cells that express TdTomato, is slightly higher than previous estimates from Jeon et al. (1998; 41%) and Schlamp et al. (2013; 51%).

Our results, like those of previous studies (Chalupa and Thompson 1980; Vaney et al. 1981), suggest that the vast majority of mouse RGCs project to the SC. The observation that ∼88% of RGCs are retrogradely labeled from the mouse SC could underestimate the true fraction of SC-projecting RGCs, however, if DiO did not robustly label axons innervating the area in which it was injected. We think this is unlikely for at least two reasons. First, >97% of the On-Off DS RGCs tuned to anterior motion, i.e., 83/85 of the GFP-expressing RGCs examined in four retinae from two Hb9::eGFP transgenic mice (Arber et al. 1999; Trenholm et al. 2011), were retrogradely labeled following injection of DiI into the SC (data not shown). Second, existing evidence supports the idea that several types of RGC avoid the SC altogether (Buhl and Peichl 1986; Gauvain and Murphy 2015; Schmidt et al. 2011; Yonehara et al. 2008).

Conversely, 88% could represent an overestimate if DiI or DiO labels cells that do not innervate the SC. We think this is unlikely for several reasons. First, as in our previous work (Gauvain and Murphy 2015), DiO was targeted to the caudal half of the SC to avoid labeling retinorecipient nuclei just rostral to the SC. Second, several results indicate that DiI/DiO taken up by SC-projecting RGCs did not spread readily to nearby cells that do not innervate the SC: *1*) neither DiI nor DiO was ever observed in amacrine cells, e.g., cells in the retina of vGlut2-Cre × Ai14 mice that lacked TdTomato (Fig. 1*A*) or cells in the retina of Chat-Cre × Ai32 mice that expressed eYFP (starburst amacrine cells; data not shown); *2*) DiO labeled <90% of vGlut2-positive/TdTomato-positive cells in the most densely labeled area of each piece of retina examined; and *3*) injecting small volumes of DiI and DiD into the medial and lateral aspects of the same SC, respectively, labeled RGCs in separate areas of the retina (Fig. 1*B*); i.e., DiI did not spread from RGCs innervating one part of the SC to RGCs innervating another part of the SC. These results indicate that the family of lipophilic dyes that includes DiO, DiI, and DiD does not spread to amacrine cells and spreads weakly, if at all, between RGCs.

Previous studies suggest that 25–50% of rodent/rabbit RGCs innervate the dLGN (Dreher et al. 1985; Linden and Perry 1983; Martin 1986; see, however, Coleman et al. 2009). More precise estimates require one to *1*) label all the dLGN-projecting RGCs *2*) without labeling any of the RGC axons that pass near (but do not synapse within) the dLGN. Our control experiments (Fig. 1C) indicate that G-deleted rabies virus satisfies only the second of these criteria; other data indicates that lipophilic dyes satisfy the first criterion but not the second.

The same technical constraints complicate efforts to determine the fraction of SC-projecting RGCs that also innervate the dLGN. It was possible, however, to determine the fraction of dLGN-projecting RGCs innervating the SC. The high percentage of dLGN-projecting RGCs retrogradely labeled from the SC (Fig. 1*D*) suggests that SC neurons have access to the vast majority of retinal input sent to the dLGN. Functional evidence (see below) suggests that the converse is unlikely.

#### Function/electrophysiology.

Retrograde transport or transgenic expression of fluorophores facilitates identification of the RGCs projecting to retinorecipient brain regions. However, in the absence of a transgenic line labeling each class of mouse RGC completely and uniquely, the only way to assay the relative abundance of different classes of RGCs innervating one or more regions is to first retrogradely label (or antidromically activate) RGCs and then characterize their responses to visual stimuli.

Many of the retrogradely labeled RGCs we characterized could be distinguished from one another on the basis of their responses to a small, relatively simple set of visual stimuli. Indeed, >80% of dLGN- and SC-projecting RGCs fell into one of the six groups we used, i.e., On-Off DS, sustained and transient Off, sustained On, transient On, and a group of On RGCs that exhibit transient responses to small-diameter stimuli. The latter group of RGCs likely includes the “W3” RGCs characterized previously (Kim et al. 2010; Zhang et al. 2012); such RGCs share some similarities to local edge detector (LED) RGCs in the rabbit retina (Levick 1967; van Wyk et al. 2006).

Categorizing cells into these groups and comparing the relative abundance of RGCs in the distinct groups revealed qualitative differences in retinal input to the dLGN and SC. In particular, On-Off DS, transient Off, transient On, and On RGCs exhibiting transient responses and small spatial receptive fields were substantially more common in the SC-projecting than dLGN-projecting population of RGCs; correspondingly, sustained Off and On RGCs comprised a higher fraction of the dLGN-projecting population of RGCs. Retinal input to the dLGN and to the SC shared at least one characteristics: On RGCs were substantially more common than Off or On-Off RGCs in both the dLGN- and SC-projecting populations. However, we know of no evidence to strongly support or dispute the idea that rabies labels different types of RGCs equally well. Likewise, we know of no study that identifies an RGC type in the mouse that preferentially synapses within the SC. Our results strongly suggest that the axons of many “W3-like” RGCs pass through the dLGN but form synapses predominantly/exclusively in the SC; we hope/expect that follow-up studies will test this hypothesis directly.

The retina contains more than the six functional classes of RGCs we described/distinguished in this study. Previous work clustered mouse RGCs into 12 distinct groups on the basis of electrophysiological responses to a panel of visual stimuli (Farrow and Masland 2011). A more recent study clustered mouse RGCs into >30 classes on the basis of responses assayed via changes in the emission of Ca^2+^-sensitive fluorophores (Baden et al. 2016); complementing physiological characterization of individual cells with analysis of the cells' somatodendritic morphology substantially increased the discriminability of different types of RGCs in that study and others like it. In our case, the density of cells labeled via GFP or mCherry was frequently too high to permit reconstruction of individual functionally characterized cells' dendritic arbors. We could have focused on areas with sparser labeling but chose, instead, to sample RGCs in densely labeled areas; this enabled a more representative sample of the classes of RGCs that “tile” (or form a mosaic in) a given area of the retina.

The class of RGC that tiles the primate retina most densely, midget RGCs, subserves high-resolution vision and projects largely, if not exclusively, to the dLGN (reviewed in Field and Chichilnisky 2007). Although the mechanisms that govern size selectivity in mouse RGCs remain unclear, our results indicate the mouse RGCs that respond best to a limited range of (generally small) stimulus sizes innervate the SC but largely avoid the dLGN. Our results further indicate that abrupt changes in light intensity produce substantially more transient changes in action potential firing in the SC-projecting pool of mouse RGCs. The first observation might explain why neurons in the primate SC and mouse dLGN generally respond best to larger stimuli. The second observation suggests that differences in the dynamics of dLGN and SC neuronal responses might be a consequence of differences in the dynamics of retinal input that neurons in the two areas receive; differences in local circuitry and/or intrinsic biophysical characteristics could, of course, also contribute to differences in the output of dLGN and SC neurons. Direct tests of these hypotheses are challenging but important; they promise to reveal how different combinations of network, synaptic, and/or cellular characteristics transform sensory signals in different neural pathways.

## GRANTS

Financial support for this work was provided by the Howard Hughes Medical Institute (HHMI). E. M. Ellis was part of the HHMI Medical Fellows program and was funded by the Foundation Fighting Blindness. B. Sivyer received support from an Australian government National Health and Medical Research Council C. J. Martin Biomedical Fellowship.

## DISCLOSURES

No conflicts of interest, financial or otherwise, are declared by the authors.

## AUTHOR CONTRIBUTIONS

E.M.E., G.G., and B.S. performed experiments; E.M.E., G.G., B.S., and G.J.M. analyzed data; E.M.E., G.G., B.S., and G.J.M. interpreted results of experiments; E.M.E., G.G., B.S., and G.J.M. edited and revised manuscript; E.M.E., G.G., B.S., and G.J.M. approved final version of manuscript; E.M.E., G.G., B.S., and G.J.M. conception and design of research; G.J.M. prepared figures; G.J.M. drafted manuscript.
